# Arete – candidate gene prioritization using biological network topology with additional evidence types

**DOI:** 10.1186/s13040-017-0141-9

**Published:** 2017-07-06

**Authors:** Artem Lysenko, Keith Anthony Boroevich, Tatsuhiko Tsunoda

**Affiliations:** 1 Laboratory for Medical Science Mathematics, RIKEN Center for Integrative Medical Sciences, 1-7-22 Suehiro-cho, Tsurumi, Yokohama, 230-0045 Japan; 2 Department of Medical Science Mathematics, Medical Research Institute, Tokyo Medical and Dental University, 1-5-45 Yushima, Bunkyo-ku, Tokyo, 113-8510 Japan; 3 CREST, JST, Tokyo, 113-8510 Japan

**Keywords:** Gene prioritisation, Biological network analysis, Cytoscape, Random walk, DIAMOnD

## Abstract

**Background:**

Refinement of candidate gene lists to select the most promising candidates for further experimental verification remains an essential step between high-throughput exploratory analysis and the discovery of specific causal genes. Given the qualitative and semantic complexity of biological data, successfully addressing this challenge requires development of flexible and interoperable solutions for making the best possible use of the largest possible fraction of all available data.

**Results:**

We have developed an easily accessible framework that links two established network-based gene prioritization approaches with a supporting isolation forest-based integrative ranking method. The defining feature of the method is that both topological information of the biological networks and additional sources of evidence can be considered at the same time. The implementation was realized as an app extension for the Cytoscape graph analysis suite, and therefore can further benefit from the synergy with other analysis methods available as part of this system.

**Conclusions:**

We provide efficient reference implementations of two popular gene prioritization algorithms – DIAMOnD and random walk with restart for the Cytoscape system. An extension of those methods was also developed that allows outputs of these algorithms to be combined with additional data. To demonstrate the utility of our software, we present two example disease gene prioritization application cases and show how our tool can be used to evaluate these different approaches.

**Electronic supplementary material:**

The online version of this article (doi:10.1186/s13040-017-0141-9) contains supplementary material, which is available to authorized users.

## Background

Identification of genes associated with a disease is an essential first step in developing novel treatments and gaining better insight into the underlying mechanisms of disease. Many widely employed contemporary experimental approaches, like genome-wide association studies (GWAS) or differential gene expression analysis, yield lists of genes potentially enriched for promising candidates [1], which then need to be further refined and verified experimentally. Network-based prioritization approaches are one of promising strategies that can effectively combine and interpret large volumes of prior knowledge about different types of interactions between biological entities. In particular, two broad strategies of network-based prioritization have emerged – those that consider global network topology by employing some type of a diffusion or Markov process formalism [2, 3] and those that focus on local topology in specific network neighborhoods [4]. Biological networks can also be further enriched by additional types of data that can potentially be used to further increase performance of network topology based methods.

Although a veritable variety of disease gene prioritization solutions are now available, the efforts so far have chiefly focused on leveraging specific, pre-defined types of data. In this respect it is possible to identify several types of typical approaches. The first approach is to develop a specialized integrated knowledgebase resource to support gene prioritization analysis. Some prominent examples in this category include PrixFixe [5], ENDEAVOR [6], GeneMANIA [7], Gene Prospector [8] and DAPPLE [9]. The advantages of such a setup is an ability to closely tailor the analysis method to make best use of these data and being able to pre-compute some of the more time-consuming analysis steps. However this comes at the cost of restricting the user’s choices and necessitates continued maintenance of the underlying datasets to ensure they remain relevant. Given the logistic constraints, access to such methods is usually delivered via web page-based interfaces [6, 8, 9] or web services [5, 7], and therefore may not be suitable for cases where confidentiality and data security is important. Approaches of the second type offer some data acquisition functionality, such as calling external web services to further enrich the input provided by the user. Tools following this strategy include Genotator, which performs real-time integration of eleven clinical genetics resources [10], GPEC, which can query different annotation databases to build up the seed set of genes [11] and JEPETTO that can dynamically retrieve additional information from pathway databases [12]. In this case, while the data can be easily kept up-to-date, the analysis approach is usually built around those specific types of data. And lastly, although some tools can work on user-provided datasets, they are only capable of using some pre-defined types of information [13, 14]. Some notable examples of such tools include NetworkPrioritizer [15] and iCTNet [16]. NetworkPrioritizer supports computation of multiple centrality measures and allows them to be combined using several rank aggregation algorithms. iCTNet is an example of a database-based approach, where a prioritization algorithm relies on a pre-integrated and developer-maintained database. In contrast to these two methods, our approach is based on similarity to a set of representative seeds and allows incorporation of both network-based and other data in the form of node annotations. Due to the extent of previous effort in disease gene prioritization tool development, only very brief summary of them could be provided here and for a more comprehensive discussion of the subject we would like to recommend the following reviews [1, 17–19].

Given potentially complex etiology of diseases and diverse types of data collected in biomedical research, we believe in potential benefits of a more flexible approach, more agnostic with respect to types of data. The benefits of such an approach would be to give greater control to the users by allowing them to make the best possible use of their own project-specific datasets as well as any publically available information. Our tool, Arete, combines network analysis capabilities with integrative analysis and in doing so allows users to further enrich these results with their own information of different types. The network-based analysis component offers two modern prioritization algorithms: random walk with restart [2] and DIAMOnD [4]. Our tool is implemented as an app plug-in for the popular Cytoscape [20] graph analysis suite in order to make the best possible use of the synergies with data acquisition and analysis capabilities of this system and its rich ecosystem of plug-ins. Our primary goal for this tool is to facilitate interactive, visual exploration of the network through means of filtering and graph annotation to direct users to sets of genes enriched for promising candidates.

### Implementation

#### Implementation of graph topology analysis methods

Arete offers two reference implementations of network-based gene prioritization approaches – a random walk with restart (RWR) [2] and DIAMOnD [4], which is an iterative, local neighborhood-based method. It was reported in [21] that diffusion-based approaches, like RWR, appear to perform better when candidate genes are somewhat dispersed throughout the network, whereas neighborhood-based approaches - when genes are concentrated in tightly linked cliques. Therefore, by offering a robust algorithm in each of these two categories Arete aims to accommodate both of these cases. In the current version only unweighted and undirected versions of both algorithms are available.

In the RWR approach, the genes (nodes in a network) are prioritized in descending order of probability of being visited by a random walker that starts from one of the seed nodes (known disease genes). Unlike several other network-based gene prioritization tools, our implementation does not use an approximate iterative solution, but rather an exact one described in [3]:

$$ {p}_{\infty }= r{\left( I- W\left(1- r\right)\right)}^{-1}{p}_0 $$

**Fig. 1:**
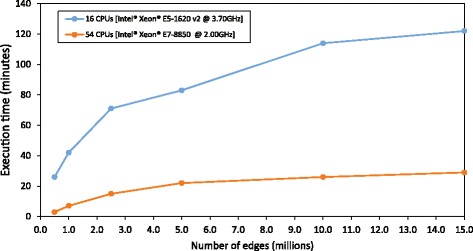
Performance evaluation of multi-threaded implementation of random walk with restart gene prioritization algorithm on systems with 16 and 54 CPU cores

Where *W* is column-normalized adjacency matrix, *r* is the probability of restart and *p* is the probability of a node to be visited by a random walker. As is evident from the formula, this approach necessitates one slow calculation step - a matrix inversion. To ensure optimal performance, we have considered eight different Java-based libraries before settling on ojAlgo [22] as an optimal solution. To evaluate the scalability of our implementation we tested it on several networks between 0.5 and 15 million edges in size (Fig. 1). All of the test networks were constructed from human co-expression data (Hsa2.v14-08) from COXPRESdb [23] by selecting edges with the highest absolute correlation until a desired number of edges was reached. On a 16-core test machine, roughly comparable to a modern high-end desktop PC, it took about 2 h to complete the calculation for the largest network. We have found that addition of extra cores offered substantial improvements, reducing time needed to process the largest test network to under 30 min. To further alleviate this issue we added an option to save and re-use pre-calculated inverted matrix from an external file. As this intermediate matrix is purely network-specific, it can be re-used with different sets of seed genes.

The DIAMOnD algorithm starts by considering the immediate neighbors of the seed nodes and selects a node with the smallest probability of having at least as many connections to seed nodes according to the hypergeometric distribution. Once a node is picked, it is added to the seed set and the process is repeated until the desired number of candidates is picked. Candidate genes are ranked in ascending order based on the iteration step at which they were picked. Both DIAMOnD and RWR network topology-based methods can be run on their own or combined with other types of data, as explained in detail in the following section. The parameter values for both of these algorithms were set according to recommendations of their respective original authors. From the analysis reported in both cases, we expect that these values are likely to be near-optimal in vast majority of cases and will rarely, if ever need to be adjusted by users.

#### Integrative prioritization approach

The integrative prioritization algorithm aims to assign a rank corresponding to how likely an unlabeled data instance is to belong to a specified class (e.g. gene being associated with a specific disease). The examples of the class instances can be specified by a user with a combination of an attribute and value that identifies a group of nodes in a Cytoscape graph. The ranking of unlabeled nodes is done with the help of an isolation random forest data structure (iRF) [24]. An isolation random forest is a collection of decision trees where each tree is constructed on a subsample of all available data. During tree construction, one attribute and one split point are selected at random at every node and the process is continued until no more splits can be done (i.e. there is only one data point left or all data points are equal). Analogous to the original method, in our implementation the construction stage of the algorithm is class label agnostic and only relies on the overall structure of the data. The scoring step is done by applying the iRF to all of the instances in the dataset. The original work used the iRF for outlier detection [24]. We found that it can also be effectively used for prioritization/ranking by incorporating target class annotation at the scoring stage as follows:

$$ score\left( k,\  c\right)={\displaystyle \sum_{t\in T}}{\displaystyle \sum_{n_i:\  k\in n}}{\left(\frac{\left|{n}_i{\displaystyle \cap } c\right|}{\left|{n}_i\right|}\right)}^{\alpha} $$

Here *k* is an instance to be scored, *c* is a set of seed instances of target class, *T* is a set of all isolation trees (*t*) in the ensemble, and *n* is a set of instances selected at a node of tree *t*. The scoring metric quantifies the co-occurrence of a particular instance with instances of a target class at different nodes. The balancing parameter, α can be adjusted to emphasize either highly specific similarity to (potentially smaller number of) seed instances (values between 0.0 and 1.0) or overall similarity to multiple seed instances (values above 1.0). The underlying rationale behind the scoring approach is that similar instances are less likely to be randomly separated and therefore will tend to co-occur at the nodes of the tree more frequently compare to unrelated ones. The advantages of proposed method include capturing effects of interacting attributes (which will generate more pure groups with higher scores), the non-parametric nature of the algorithm and relatively few critical options requiring input from the user.

The GUI interface offers three customization options: number of trees to generate, balancing parameter and a switch for controlling how to select a value for splitting at tree nodes. As the algorithm is stochastic, selecting a larger number of trees will tend to lead to more consistent results between different runs at the cost of lower speed, though the underlying level of performance will only be adversely affected if this option is set very low. By default, the split point can be any number between the minimum and maximum values of an attribute in a set of instances selected for particular node during construction stage. This default behavior can be changed to make all splits equally likely, which is equivalent to rank-transforming all data. The default values for all these parameters were chosen by performing tests on gene sets for particular diseases and Gene Ontology biological process categories between 10–100 genes in size. The sets of reference disease genes for this task were taken from DisGeNET database [25] and were chosen to be distinct from the ones used in the evaluation example described below. The aim was to set all parameter values at levels where an adequate result will be generated in most cases in order to create a reasonable starting point from which a user can experiment further.

#### Cytoscape App user interface

**Fig. 2:**
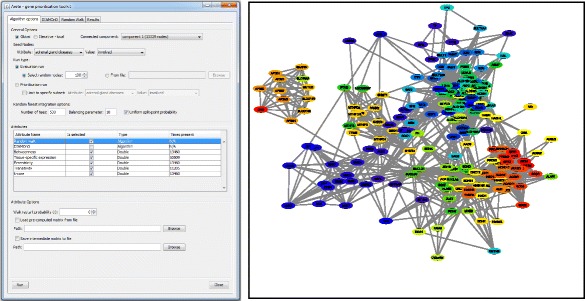
The main interface of the Arete Cytoscape app (*left*) and visualization of DIAMOnD gene ranking generated using annotation and filtering functionality of the app (*right*)

We have implemented a graphical user interface for Arete and packaged it as an app for the Cytoscape system (Fig. 2 – left panel). The interface consists of four tabs, one each for DIAMOnD and RWR topology-based gene prioritization methods, integrative prioritization and exploration of results. When the app is loaded, an active graph in Cytoscape is automatically scanned for applicable attributes and a connected components analysis is performed to identify meaningful subsets. The attributes can be selected for inclusion in the analysis by selecting them in the main panel. Individual tabs for DIAMOnD and RWR are provided in order to simplify access to these algorithms and only show options relevant to them, whereas the main Arete tab allows more complex analysis runs to be configured. In particular, it allows configuring either evaluation or prioritization algorithm runs or performing integrative analysis over multiple node attributes in combination with graph topology-based methods.

During evaluation, each known “true positive” gene that is withheld from the seed set for a given evaluation run is ranked in its own list of reference unlabeled genes. The tool offers two options for providing such reference lists. The first (default) option is to automatically construct these lists by drawing random non-seed genes from the network. This option is the likely most common application scenario, where a user does not have a pre-defined reference set of interest. The second option is for user to provide their own reference lists. This is done by providing a separate tab-delimited file where the first column is a relevant gene and the rest of the line is its reference list. This option has been used in the first example use-case, where a reference list was constructed using neighboring genes in the genome.

Lastly, the prioritized genes can be highlighted in the Cytoscape network view by changing the color of respective nodes according to their ranks (Fig. 2 – right panel). A filter can be applied to select highly ranked nodes and, optionally, their neighbors at a particular level.

#### Example use-case 1: ranking candidate genes in a genomic region

For this use-case, we have applied our approach in a simulated scenario of analyzing GWAS data by extending an example dataset that was used in original DIAMOnD evaluation study [4]. This dataset integrated several high-confidence sources of gene and protein interactions and a set of genes for 69 different diseases. To complement it, we constructed a control set of unlabeled genes for each of the disease-associated seed genes by taking 100 closest neighboring genes in the genome, which were also present in the network and were not seeds themselves. This evaluation setup is analogous to the one used in the original RWR paper [2]. To demonstrate how multiple types of data can be incorporated into this analysis, we have compiled a list of five additional gene-specific property metrics – four graph-theoretic properties and one relating to tissue specific patterns of expression. These properties are described in Table 1. Multiple previous studies [26–29] have reported that distributions of various graph centrality measures can be significantly different between sets of disease-associated and unrelated genes. It has also been shown to be true for specific patterns of gene expression, where disease-related genes tend to be non-ubiquitously expressed across different tissues [30]. To capture this aspect we downloaded tissue-specific expression patterns from the Human Protein Atlas resource [31], which has profiled expression of 86% of all human proteins across 83 different cell types. The tissue-specific expression property was calculated as a proportion of all tissues where at least “low” level of expression was reported in the Human Protein Atlas database.

**Table 1:** Additional metrics used for integrative analysis example and informal descriptions of what properties they capture

Metric name	Property captured
Eccentricity	Overall remoteness from all other nodes
Transitivity	Density of interlinks among immediate neighbors
Betweenness	Network “choke points” with high proportion of shortest paths going through them
Tissue-specific expression	Ubiquitous versus tissue-specific expression
k-core number	Location in a dense core versus network periphery

The comparative evaluation considered five different setups. First, the prioritization was done separately using RWR, DIAMOnD and iRF based on the five metrics only. Then, two more runs where performed where DIAMOnD or RWR scores were also included as features in the iRF set. To explore the results we have computed ROC-AUC statistic according to the method described in [2] using leave-one-out, 3-fold and 5-fold cross-validation schemes. Additionally, we have looked at fold-enrichment for known disease genes in different quartiles of resulting ranked lists. To provide a representative sample of likely performance of all methods in a “worst case”/baseline scenario, all reported analyses were done with the chosen default options of our application. Therefore, no attempt was made to specifically optimize setting for each of the diseases in the example dataset. As associated gene sets are likely to be quite distinctive, we expect that different parameters may be optimal in each individual case. In practice, an expected use-case will only usually involve a single disease or set of genes and a user may choose to interactively optimize the settings to further improve results.

#### Example use-case 2: ranking candidate genes in a transcriptomic study

For the second example we illustrate how our software can be used in combination with an example transcriptomic study. Here we have used data from a microarray profiling experiment E-GEOD-15245, which investigated how gene transcription in the blood changes in the period preceding multiple sclerosis (MS) relapse [32]. A complete, processed dataset from this study was downloaded from the EBI’s ArrayExpress database [33]. We have chosen samples taken less than a year prior to observed MS relapse and where “definite MS” was confirmed. The reasoning behind this was that these samples are most likely to capture disease-relevant responses and therefore will be most useful for identification of disease-driving genes. These selected 24 expression profiles were scaled and integrated with the network and a known set of MS genes. Both the network and MS gene set were taken from the dataset used in the first use-case. The combined dataset was again analyzed using all of the methods available in Arete tool with all relevant parameters left at recommended default values. In this case we have chosen to evaluate the performance by drawing 100 random reference genes (per each known MS gene) from all unlabeled genes in the network, as not having a pre-defined reference gene list is more consistent with an expected scenario for transcriptomics-based application cases.

## Results and discussion

At the time of writing, we were aware of three tools that offer different variants of the random walk algorithm for the Cystoscope suite, however, all of these offered an approximate, iterative solution rather than an exact one. One of the advantages of the exact solution is that it has been shown to be robust to restart probability parameter [2] and therefore will produce a near-optimal result without the need for time-consuming optimisation. At present, Arete is also the first tool to provide an implementation of DIAMOnD algorithm in Cytoscape. In terms of providing the evaluation functionality, the only other tool also offering it is GPEC, but GPEC has somewhat limited dataset customization functionality and is no longer available for Cytoscape 3.0 or later. As we outlined in the introduction, with respect to integrative analysis, the diversity of data and integration methods being used is quite extensive. However, the main focus of most efforts has so far been to optimally exploit particular public datasets, or to closely couple the analysis method with specific, pre-generated datasets. To the contrary, our intention has been to develop an approach that is flexible and generic. In combination with the easy-to-use data import and acquisition methods of Cytoscape system our approach allows users to build and leverage their own resources. Additional flexibility is achieved by: (1) offering performance evaluation capabilities that can be used to explore and understand the impact of particular features and (2) interactive, user-driven exploration of results in the graph interface.

**Fig. 3:**
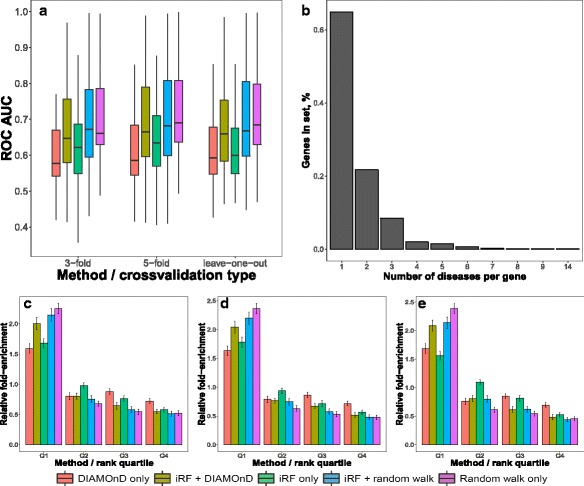
Comparison of different gene prioritization approaches offered in Arete app. **a** Box plot of ROC AUC scores of leave-one-out, 3-fold and 5-fold cross-validation for 69 different sets of disease-related genes. **b** Percentages of genes associated with multiple diseases in our reference set. Bottom row shows corresponding fold-enrichment statistics for the four quartiles of ranked gene lists profiled using 3-fold (**c**), 5-fold **d** and leave-one-out **e** cross-validation schemes

**Fig. 4:**
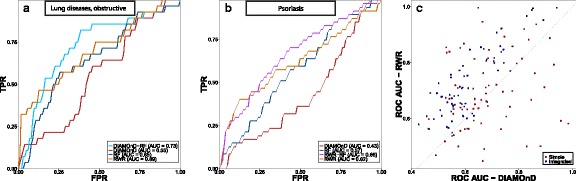
**a** and **b** show example ROC curves for two diseases – obstructive lung disease and psoriasis, respectively. **c** Comparison of ROC-AUC scores for 69 individual diseases using DIAMOnD and RWR approaches. Scores of canonical versions are shown in blue and scores where those methods were combined with additional data using iRF approach are shown in red. Each point represents a set of genes for particular disease

The first evaluation example has shown that our proposed integration method can be successfully used with network topology-based features to improve results (Fig. 3). In particular, when combined together, DIAMOnD and integrative prioritization (iRF) performed substantially better relative to when these approaches where used in isolation (Fig. 3a). It can be seen in Fig. 3a that according to our evaluation, the RWR method performed best overall, however the picture becomes more complicated when ranking of individual diseases by different methods is examined (Fig. 4c). As diseases are highly heterogeneous, it is to be expected that different network-based properties and expression patterns will not have the same importance in all of the cases. Therefore, we have observed cases where either of the two network-based methods has performed best (Fig. 4a and b). Similarly, when DIAMOnD is compared with RWR without additional data, it did outperform RWR in 16 out of 69 cases, which is consistent with what was previously reported in [4]. These results highlight potential benefits of having access to several distinctive network-based prioritization approaches and alternative perspectives they offer.

In addition to ROC-AUC analysis, we have also looked at the fold-enrichment, which, again, was explored using 3-fold, 5-fold and leave-one-out cross validation schemes (Fig. 3c-d). For this analysis we have split the ranked lists into four quartiles and compared the actual distribution of known disease genes with the one expected by chance. For all of the methods, a substantially higher enrichment was predominantly achieved in the first quartile, where between 1.6 and 2.4-fold more relevant genes were recovered.

Disease-related genes can play a role in more than one disease and are often associated with high network centrality, which is emphasized both by incorporation of network-specific properties via iRF and by the RWR algorithm. Potentially, this can cause a positive bias with respect to those genes, as inevitably there will be some overlap between sets of genes for different diseases and high centrality genes are more likely to be in this overlap. To explore this possibility, we have looked at the distribution of multi-disease genes in our dataset (Fig. 3b) and investigated whether such effects had substantial influence on performance (Additional file 1: Figure S1) As about 67% of all genes in our dataset were only involved in one disease, we have split our data into a single-disease and multi-disease subsets (2 or more associated diseases per gene) and re-calculated all of the performance statistics for these subsets. Although the performance was slightly higher for multi-disease genes according to both ROC- AUC and fold-enrichment metrics, this difference was too small to indicate a definite and substantial bias in this case (Additional file 1: Figure S1).

**Fig. 5:**
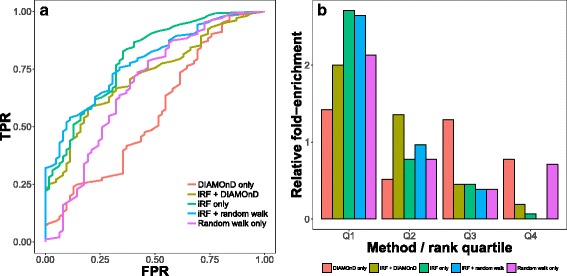
Performance evaluation of Arete methods on transcriptomics data, which profiled relapse during multiple sclerosis progression. **a** ROC-AUC curves; **b** fold-enrichment for each quartile of a reference list

The example prioritization of MS-related genes using transcriptomics data has shown that our method can be effectively used with such data to identify promising disease-associated genes (Fig. 5a-b). In this case, the iRF used on transcriptomics data has produced an overall highest fold-enrichment in the first quartile and the best ROC-AUC. DIAMOnD algorithm has not performed well in this case (ROC-AUC of 0.562) and, in contrast to the overall trend from use-case 1, no benefit was observed from combining it with transcriptomics data via iRF (ROC-AUC of 0.722), as the overall performance was still lower than iRF alone (ROC-AUC of 0.796). Random walk with restart performed substantially worse than iRF (ROC-AUC 0.678). In combination with iRF it has produced a comparable ROC-AUC score (0.783) and therefore did not lead to a substantial decline in performance relative to iRF, as was the case for iRF + DIAMOnD.

To conclude, as previously noted in [21], our results from use-case one also hint at the possibility that at least some of predictive network-based properties may be particularly effective only in specific cases and consequently heterogeneity likely exist between such properties of genes associated with different diseases. The second use-case illustrated how our approach can be used to identify most relevant disease-causing genes from transcriptomics data. These results indicate that even without further optimization, all of the methods provided in Arete can be suitable for identifying approximately relevant gene sets from experimental data. Therefore, in combination with interactive visualization capabilities of the Cytoscape system itself, Arete can effectively support analysis of complex biological networks by facilitating identification of smaller, meaningful gene sets for further manual exploration by the user.

## Conclusion

Although large and diverse number of disease gene prioritization software are now available, emphasis has been primarily on approaches that either work on a specific pre-integrated knowledgebases or public web resources; or are only able to consider particular types of biomedical data by design. At the same time, biomedical application cases often rely on their own ‘omics datasets, data from different studies and experiments and highly specialized expert knowledge. This creates a niche for a more generalized tool that can allow non-technical users to exploit project-specific integrated datasets, identify promising combinations of predictive features and find likely candidate genes, which are more directly supported by context-specific evidence. Our proposed solution fills this niche by achieving a pivot between flexibility and ease-of-use, while at the same time also delivering adequate levels of performance and evaluation capabilities for comparing different setups. Using the example analysis presented in this paper, we also demonstrated that our proposed multiple evidence integration method can further enhance the performance achievable by network topology-based methods alone.

## Additional file

Additional file 1: Figure S1. Evaluation of performance for multi-disease and single-disease gene subsets. (PDF 183 kb)
