# Supplementary material for: Arete – candidate gene prioritization using biological network topology with additional evidence types
Source: BioData Min. 2017 Jul 6;10:22. doi: 10.1186/s13040-017-0141-9 (PMC5501438; doi:10.1186/s13040-017-0141-9)
Supplement: Additional file 1: Figure S1. — Evaluation of performance for multi-disease and single-disease gene subsets. (PDF 183 kb) [file 13040_2017_141_MOESM1_ESM.pdf]

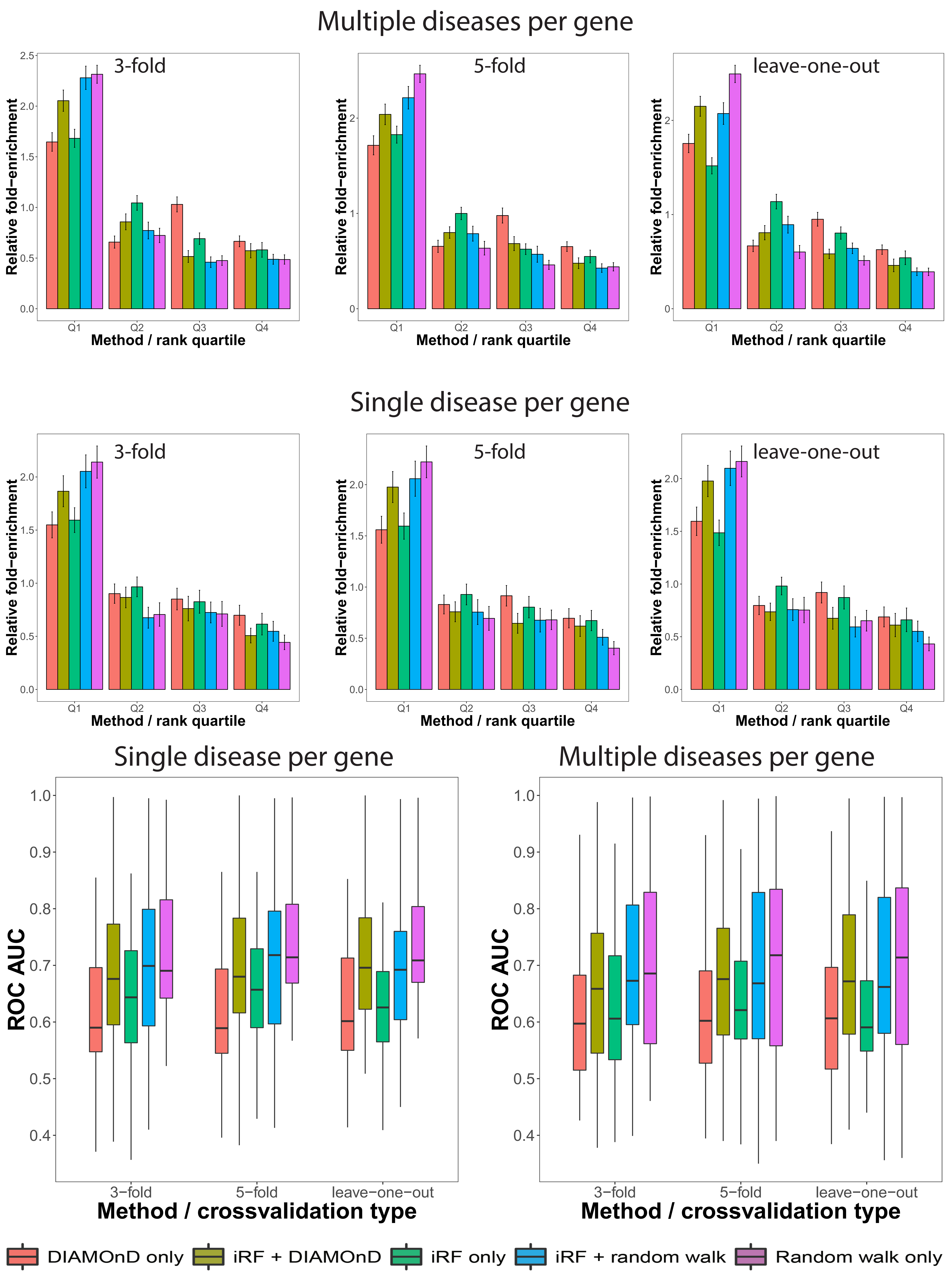
Multiple diseases per gene
3-fold
5-fold
leave-one-out
Relative fold−enrichment
Method / rank quartile
Q1
Q2
Q3
Q4
Single disease per gene
3-fold
5-fold
leave-one-out
Relative fold−enrichment
Method / rank quartile
Q1
Q2
Q3
Q4
Single disease per gene
Multiple diseases per gene
ROC AUC
Method / crossvalidation type
3−fold
5−fold
leave−one−out
DIAMOnD only
iRF + DIAMOnD
iRF only
iRF + random walk
Random walk only
